# Expression of the Metalloproteinase ADAM8 Is Upregulated in Liver Inflammation Models and Enhances Cytokine Release *In Vitro*

**DOI:** 10.1155/2021/6665028

**Published:** 2021-03-11

**Authors:** Tanzeela Awan, Aaron Babendreyer, Justyna Wozniak, Abid Mahmood Alvi, Viktor Sterzer, Lena Cook, Jörg W. Bartsch, Christian Liedtke, Daniela Yildiz, Andreas Ludwig

**Affiliations:** ^1^Institute of Molecular Pharmacology, RWTH Aachen University, Aachen, Germany; ^2^Department of Medicine II, University Hospital RWTH Aachen, Aachen, Germany; ^3^Department of Neurosurgery, Philipps University of Marburg, University Hospital Marburg, Marburg, Germany; ^4^Department of Medicine III, University Hospital RWTH Aachen, Aachen, Germany; ^5^Institute of Experimental and Clinical Pharmacology and Toxicology, PZMS, ZHMB, Saarland University, Homburg, Germany

## Abstract

Acute and chronic liver inflammation is driven by cytokine and chemokine release from various cell types in the liver. Here, we report that the induction of inflammatory mediators is associated with a yet undescribed upregulation of the metalloproteinase ADAM8 in different murine hepatitis models. We further show the importance of ADAM8 expression for the production of inflammatory mediators in cultured liver cells. As a model of acute inflammation, we investigated liver tissue from lipopolysaccharide- (LPS-) treated mice in which ADAM8 expression was markedly upregulated compared to control mice. *In vitro*, stimulation with LPS enhanced ADAM8 expression in murine and human endothelial and hepatoma cell lines as well as in primary murine hepatocytes. The enhanced ADAM8 expression was associated with an upregulation of TNF-*α* and IL-6 expression and release. Inhibition studies indicate that the cytokine response of hepatoma cells to LPS depends on the activity of ADAM8 and that signalling by TNF-*α* can contribute to these ADAM8-dependent effects. The role of ADAM8 was further confirmed with primary hepatocytes from ADAM8 knockout mice in which TNF-*α* and IL-6 induction and release were considerably attenuated. As a model of chronic liver injury, we studied liver tissue from mice undergoing high-fat diet-induced steatohepatitis and again observed upregulation of ADAM8 mRNA expression compared to healthy controls. *In vitro*, ADAM8 expression was upregulated in hepatoma, endothelial, and stellate cell lines by various mediators of steatohepatitis including fatty acid (linoleic-oleic acid), IL-1*β*, TNF-*α*, IFN-*γ*, and TGF-*β*. Upregulation of ADAM8 was associated with the induction and release of proinflammatory cytokines (TNF-*α* and IL-6) and chemokines (CX3CL1). Finally, knockdown of ADAM8 expression in all tested cell types attenuated the release of these mediators. Thus, ADAM8 is upregulated in acute and chronic liver inflammation and is able to promote inflammation by enhancing expression and release of inflammatory mediators.

## 1. Introduction

The liver is the main site for the metabolism of lipids, proteins, and carbohydrates and is constantly exposed to gut-derived bacterial metabolites such as lipopolysaccharides (LPS) and toxins. These metabolites are able to trigger immune reactions in the liver resulting in acute inflammation response [1]. LPS is immediately sensed by various liver cell types and initiates an inflammatory response by triggering the production of inflammatory cytokines *via* activation of the Toll-like receptor 4 (TLR4) signalling pathway [2] leading to the systemic inflammatory syndrome [3]. The combined effect of LPS with other liver pathologies also worsens the outcome of liver damage resulting in chronic liver inflammation. Other liver pathologies that may cause chronic liver inflammation include hepatic viral infections, metabolic disorders, obesity, chronic alcohol consumption, and autoimmune disorders. Fatty liver is the most common form of chronic liver disease. Generally, nonalcoholic fatty liver disease (NAFLD) is a spectrum of chronic liver disease characterized by excessive cytoplasmic retention of triglyceride which is the major consequence of metabolic stress. This condition ranges from simple nonalcoholic fatty liver (NAFL) to progressive nonalcoholic steatohepatitis (NASH) which can further develop into liver fibrosis and cirrhosis and/or hepatocellular carcinoma (HCC) [4]. Hepatocytes are activated by fatty acid and interleukin 1beta (IL-1*β*) leading to activation of the nuclear factor kappa B (NF-*κ*B) pathway. This induces the gene transcription of several proinflammatory cytokines and chemokines such as tumour necrosis factor-alpha (TNF-*α*), IL-6, and CC chemokine ligand 2 (CCL2) [5, 6].

Proteolytically active members of a disintegrin and metalloproteinase (ADAM) family are predominantly involved in cleavage and shedding of various membrane-bound proteins such as cytokines, cytokine receptors, growth factors, and adhesion molecules [7]. ADAMs are considered as key regulators of inflammatory processes [8, 9]. The metalloproteinase ADAM8 is a proteolytically active member of this family, which is upregulated in several cancers and inflammatory diseases, where ADAM8 can act as a protease cleaving surface molecules and as an adhesion molecules to promote cell migration. A proinflammatory role of ADAM8 has been described in a few inflammatory disorders including acute lung inflammation and asthma [10–13]. However, other studies have also identified anti-inflammatory activities in asthma and COPD [14]. Therefore, it is worthy to understand the contribution of ADAM8 also in liver inflammation. We and others have recently described the upregulation of ADAM8 in hepatocellular carcinoma [15, 16]. In malignant diseases, ADAM8 can promote prometastatic cell movement independently of its proteolytic activity [15–17]. However, ADAM8 has not been yet studied in the context of acute and chronic liver inflammation. Here, we report the yet undescribed upregulation of ADAM8 in different murine models of LPS or high-fat-induced liver inflammation and its importance for the production of inflammatory mediators in different types of cultured murine and human liver cells.

## 2. Materials and Methods

### 2.1. Antibodies and Reagents

Rabbit monoclonal antibody (mAb) against human/mouse ADAM8 (EPR14612) (N-terminus) was from Abcam. Mouse mAb against GAPDH (GA1R) was from Thermo Scientific (Waltham, MA, USA). Rabbit polyclonal anti-ADAM8 antibody was from Lifespan (Biosciences, Washington, USA). Peroxidase- (POD-) conjugated secondary antibodies were from Jackson Immuno Research (Hamburg, Germany). All antibodies were applied as per manufacturer's instructions. Lipopolysaccharide (LPS) for *in vitro* use was from *E. coli* 0127:B8 and purchased from Sigma-Aldrich (Munich, Germany). LPS used *in vivo* was from Sigma-Aldrich (Steinheim, Germany). Fatty acid was a combination of linoleic acid, oleic acid, and albumin (100x Sigma Aldrich, Steinheim, Germany). Recombinant murine and human TNF-*α*, IFN-*γ*, IL-1*β*, and TGF-*β* were from PeproTech GmbH (Hamburg, Germany). Infliximab was from Sigma-Aldrich (St. Louis, USA). The hydroxamate-based inhibitors marimastat (BB-2516) and batimastat (BB-94) were from Merck, Calbiochem (Darmstadt, Germany); the cyclic peptide BK-1361 was synthesized by Peptide 2.0 (Chantilly, VA, USA).

### 2.2. Murine Tissue Samples

All murine liver tissues used for this study were derived from previous published studies. The mRNA had been isolated from the liver tissue and stored immediately at -80°C. The mRNA was then analysed in this study. The liver tissues were obtained from the murine nonalcoholic fatty liver disease (NAFLD) model (mice fed on a high-fat diet (HFD) for 7 weeks and 14 weeks) and respective controls as described before [18].

The mouse liver tissues from liver injury models include lipopolysaccharide (10 *μ*g) and D-galactosamine (700 mg) (LPS/GalN-induced liver injury) (6 h), bile duct ligation (BDL) model (3 weeks), and partial hepatectomy (PH) model (48 h) as described before [19].

For the LPS model of acute liver injury, 8-10-week-old male wild-type Bl6 mice had been used as described previously [20]. Briefly, mice were injected i.p. with LPS (15 *μ*g/100 ml) or 0.9% NaCl as control. After 6 h, the mice were euthanised and sacrificed. Treatment and organ sampling had been approved for previous studies by the authority for environment conservation and consumer protection of the state North Rhine-Westphalia (State Agency for Nature, Environment and Consumer Protection, LANUV, Recklinghausen, Germany) (81-02.04.2019.A060).

### 2.3. Cell Culture

Primary murine hepatocytes were freshly isolated from C57BL/6J mice as described [21] and cultured in William's E medium supplemented with 1% L-glutamine, 10% fetal calf serum, and 1% penicillin/streptomycin. The origin of murine Hepa1-6 and human HepG2 hepatoma/hepatoblastoma cell lines and the murine LSEC and human EA.hy926 endothelial cell lines of murine GRX and human LX2 stellate cell lines has been described elsewhere [22–24]. Cells were regularly controlled for typical morphology and responsiveness to typical stimulators detailed in the experiments. All human and murine cell lines were cultured in DMEM supplemented with 10% fetal calf serum and 1% penicillin/streptomycin (all from Sigma-Aldrich).

### 2.4. Transfection of siRNA

Murine Hepa1-6 hepatoma cells were transfected with two different ADAM8 stealth siRNA nucleotides (83234478) or control stealth siRNA oligonucleotides (12935300) (Eurogentec, Liège, Belgium), using Lipofectamine RNAiMAX (Invitrogen, Germany) according to the manufacturer's instructions. Briefly, 2 × 10^5^ cells were seeded in six-well plates in complete medium and subsequently transfected with the respective siRNA. The siRNA silencing effect was analysed 96 h after transfection by qPCR and Western blot.

### 2.5. Lentiviral Transduction

Short hairpin RNA (shRNA) targeting ADAM8 was inserted into the lentiviral expression vector pLVTHM (Addgene plasmid 12247) as described [25]. The targeting sequences were agagaaggtttgctggaaa and gctgctgttctaacctcag. The sequence ccgtcacatcaattgccgt served as control shRNA (shRNA_ctrl). Successful and efficient transduction was assessed by green fluorescence protein (GFP) expression encoded by pLVTHM. The silencing effect was analysed 72-96 h after transduction by qPCR and Western blot [25].

### 2.6. Quantitative PCR

The mRNA expression levels of target genes were measured by quantitative polymerase chain reaction (qPCR) as described [13] and normalised to that of glyceraldehyde-3-phosphate dehydrogenase (GAPDH) for human cells and to ribosomal protein S29 (*Rps29*) for murine cells. For murine liver tissues, *Gapdh*, *18S*, *β*-*actin*, and *Rps29* were used as reference genes after validation by the geNorm algorithm as described [26], and accordingly, average of two or three of these reference genes was used to normalise the target gene expression. RNA was extracted using RNeasy Kit (Qiagen, Hilden, Germany) and quantified (NanoDrop, Peqlab, Erlangen, Germany). RNA (1 *μ*g from tissues and 500 ng from cells) was reverse-transcribed using PrimeScript RT Reagent Kit (Takara Bio Europe, Saint-Germain-en-Laye, France) according to the manufacturer's protocols. PCR reactions were then performed in duplicates of 10 *μ*l volume containing 1 *μ*l of cDNA template, 5 *μ*l 2x LightCycler SYBR Green Master Mix: SYBR Premix Ex Taq II (Takara, Fitchburg, WI, USA), 3 *μ*l H_2_O, and 0.5 *μ*M forward and reverse primer. Primers and PCR cycles are specified in the supplements (Table S1). All PCR reactions were run on a LightCycler® 480 System (Roche, Basel, Switzerland). Standard curves for target genes and reference genes were prepared from a serial dilution of pooled cDNA products of all samples. Data were obtained as the crossing point value normalised according to the E-method using the LightCycler® 480 software 1.5 (Roche, Basel, Switzerland).

### 2.7. Immunohistochemistry

Immunohistochemical analysis of 5 *μ*m thick paraffin-embedded liver tissue sections using a rabbit polyclonal anti-ADAM8 antibody (1 : 200 dilution) was performed as previously described [16]. All stained microscopic images were taken at magnification of ×200 with a Zeiss Axio Imager Z1 microscope, AxioCam MRm, and HRc cameras using AxioVision 4.8 software (Carl Zeiss, Oberkochen, Germany).

### 2.8. Western Blotting

Western blotting was performed as described [13]. 300 *μ*l lysis buffer (20 mM Tris-HCl, 150 mM NaCl, 1% Triton X-100, 1 mM EDTA, 1 mM Na_3_VO_4_, 1 mM PMSF, 10 mM 1,10-phenanthroline monohydrate, and 1-fold complete inhibitor (Roche)) was used to lyse the cultured cells (1 × 10^6^). After 10 min incubation with lysis buffer, the cells were centrifuged at 16,000 *g* for 5 min. Afterwards, cell lysates containing 15-20 *μ*g protein (analysed by bicinchoninic acid assay, Thermo Fisher/Pierce) were heated in SDS-reducing sample buffer (250 mM Tris HCl (pH 6.8), 50% (*w*/*v*) glycerol, 10% (*w*/*v*) SDS, 0.1% bromophenol blue, and 5% *β*-mercaptoethanol) and subjected to SDS-polyacrylamide gel electrophoresis using 10% Tris-glycine gels. Proteins were then transferred onto polyvinylidene difluoride (PVDF) membranes (Hybond P, Amersham; 10600023). Membranes were blocked with 5% (*w*/*v*) nonfat dry milk in Tris-buffered saline with 0.05% Tween. Membranes were probed overnight with primary antibodies followed by incubation with POD-coupled secondary antibodies (diluted 1 : 20,000). After addition of chemiluminescence substrate (ECL advanced, Amersham), signals were recorded using luminescent image analyser LAS3000 (Fujifilm, Tokyo, Japan) and quantified using open-source ImageJ software (Wayne Rasband, NIH). The specificity of the ADAM8 antibody was shown by comparison to siRNA- and shRNA-treated cells as well as murine primary hepatocytes of wild-type and knockout animals.

### 2.9. ELISA

The amounts of human/murine TNF-*α*, human/murine IL-6, human IL-8 or murine KC, human/murine chemokine CX3C motif ligand 1 (CX3CL1/fractalkine), and human/murine chemokine CXC motif ligand 16 (CXCL16) into the supernatant were analysed by ELISA as described [27]. Before the measurement, the culture supernatants were concentrated from 3 ml to 0.5 ml using Vivaspin 6 columns (10.000 MWCO) (Sartorius, Göttingen, Germany). ELISA was performed according to the manufacturer's protocols using the provided standard proteins. The absorption was analysed with the FLUOstar OPTIMA microplate reader (BMG Labtech, Ortenberg, Germany).

### 2.10. Statistics

Statistical analysis was performed as described before [28]. Briefly, raw data from at least three independent experiments were analysed by general mixed model analysis (PROC GLIMMIX, SAS 9.4, SAS Institute Inc., Cary, USA) and assumed to be derived from either normal or log normal distributions. Residual plots and the Shapiro-Wilk test were used as diagnostics. If necessary, the day of experiment conduction was set as random to assess differences in the size of treatment effects across the results. According to the COVTEST statement, all datasets were homoscedastic. Multiple comparisons were corrected by false discovery rate (FDR).

## 3. Results

### 3.1. ADAM8 Is Overexpressed in the Murine Model of LPS-Induced Acute Liver Inflammation

**Figure 1:**
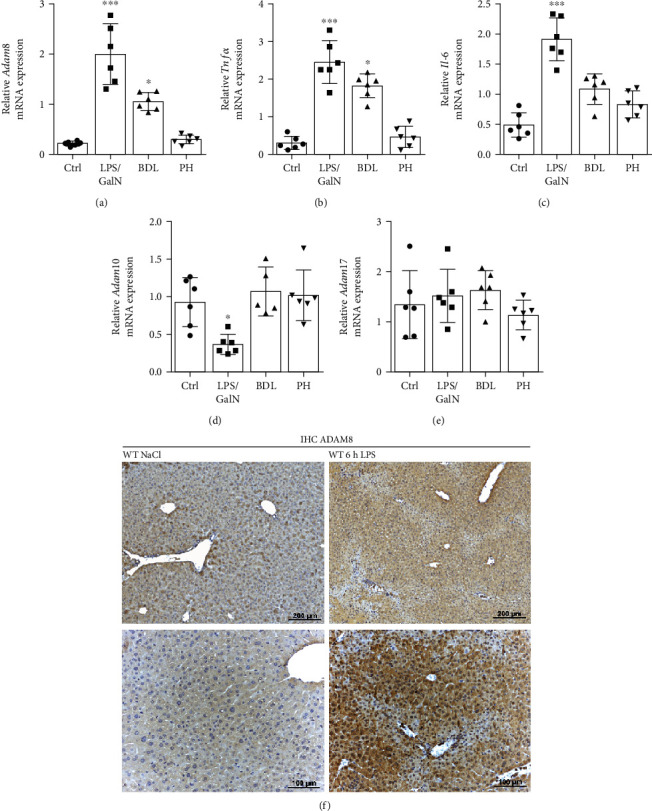
Upregulation of ADAM8 mRNA expression in different murine models of liver injury. (a–e) Liver tissue was obtained from control mice (Ctrl) or mice with different types of liver injury induced by either treatment with LPS/GalN for 6 h, by bile duct ligation (BDL) (3 weeks), or by partial hepatectomy (PH) (48 h). The mRNA expression of *Adam8* (a), *TNF-α* (b), *IL-6* (c), *Adam10* (d), and *Adam17* (e) in these livers was determined by qPCR. The mRNA expression was normalised to the average of two reference genes: *Rps29* and *Gapdh*. Data are shown as the mean ± SD. Six mice were used in each group. Differences to the control are indicated as asterisks (^∗^*p* < 0.05, ^∗∗^*p* < 0.01, and ^∗∗∗^*p* < 0.001). (f) Immunohistochemistry analysis of liver sections from mice treated with or without LPS for 6 h with a polyclonal antibody against ADAM8. Representative tissue stainings for two different mice per group (*n* = 3) are shown.

Mouse liver tissues from different acute liver injury models were analysed for their mRNA expression of ADAM8, ADAM10, and ADAM17. Additionally, the mRNA expression of the key proinflammatory cytokines TNF-*α* and IL-6 was measured to evaluate the extent of inflammation in each model. The liver injury models included LPS/GalN-induced liver injury, bile duct ligation- (BDL-) induced liver injury, and partial hepatectomy- (PH-) induced liver injury. The ADAM8 mRNA expression was clearly elevated in LPS/GalN-induced liver injury and moderately induced in the BDL-induced model compared to healthy controls (Figure 1(a)). A similar trend in mRNA expression was seen for TNF-*α* and IL-6 in LPS/GalN model and for TNF-*α* in the BDL model (Figures 1(b) and 1(c)). Of note, PH did not significantly induce ADAM8 expression. In addition, 48 h after PH, induction of TNF-*α* and IL-6 was no longer visible, which is in good agreement with previous reports [29]. In contrast to ADAM8, the related proteases ADAM10 and ADAM17 were not upregulated in the investigated models (Figures 1(d) and 1(e)), which is consistent with the general notion that the latter proteases are abundantly expressed at a high level and not markedly further regulated on the transcriptional level under inflammatory conditions. The increase in ADAM8 mRNA expression was also observed in the mouse livers only treated with LPS for 6 h (Figure S1A). In addition, tissue sections of these mice were investigated by immunohistochemistry (Figure 1(f)). Basal immunoreactivity for the used anti-ADAM8 antibody was noted predominantly in hepatocytes from untreated mice. This reactivity was overall stronger in hepatocytes from LPS-treated mice but varied among the investigated animals. At this early time point, no histological signs of liver inflammation were observed. These observations suggest that ADAM8 expression can be effectively induced *in vivo* by LPS. This induction correlates with the gene induction of other inflammatory mediators such as TNF-*α* and IL-6 and precedes cellular events of inflammation.

### 3.2. LPS Treatment Enhances ADAM8 Expression in Hepatoma Cells and Endothelial Cells

**Figure 2:**
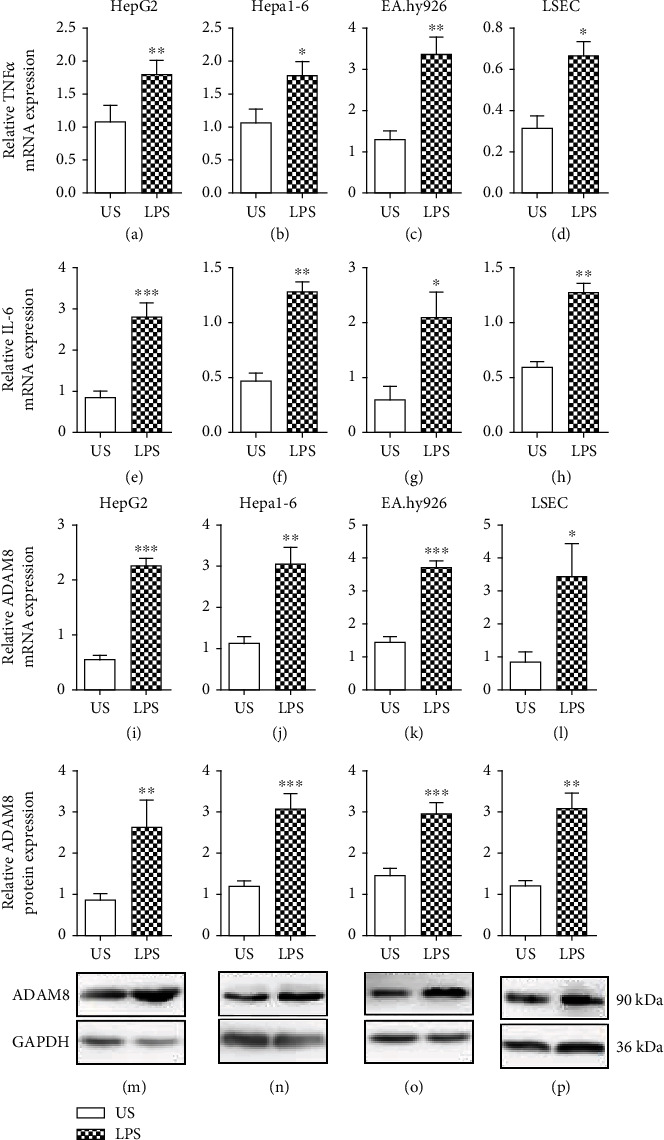
Upregulation of ADAM8 expression in hepatocyte and endothelial cells by LPS. (a–p) Human HepG2 and murine Hepa1-6 hepatoma cells as well as human EA.hy926 and murine LSEC endothelial cells were stimulated with or without LPS (1 *μ*g/ml). After 24 h of stimulation, cells were analysed for mRNA expression of TNF-*α* (a–d), IL-6 (e–h), and ADAM8 (i–l). The mRNA expression level was determined by qPCR and normalised to the mRNA expression of GAPDH (for human cells) and *Rps29* (for murine cells). Additionally, ADAM8 protein expression was detected by Western blot analysis (m–p). The antibody detects a band of approximately 90 kDa, i.e., mature ADAM8. The data are shown as representative blot for each cell line. The band intensities were quantified by densitometry and normalised to GAPDH protein expression. Quantitative data are shown as the mean + SD of 3-4 independent experiments. Significant differences to the unstimulated controls are indicated by asterisks (^∗^*p* < 0.05, ^∗∗^*p* < 0.01, and ^∗∗∗^*p* < 0.001).

To study coregulation of ADAM8 and inflammatory cytokines by LPS treatment in an *in vitro* setting, we used human and murine hepatocyte cell lines (HepG2 and Hepa1-6, respectively) as well as human and murine endothelial cell lines (EA.hy926 and LSEC, respectively). Stimulation with LPS for 24 h clearly enhanced the mRNA expression of TNF-*α* and IL-6 in both hepatoma and both endothelial cell lines (Figures 2(a)–2(h)). This was associated with increased mRNA expression of ADAM8 by these cells (Figures 2(i)-2(l)). To confirm ADAM8 upregulation at the protein level, cell lysates were analysed by Western blotting (Figures 2(m)–2(p)). A protein band was detected at 90 kDa corresponding to the mature form of ADAM8 as previously reported [16]. This band was clearly more prominent when hepatoma cells or endothelial cells were stimulated with LPS confirming the upregulation of mature and active ADAM8 on the protein level.

### 3.3. Cytokine Release from LPS-Treated Hepatoma Cells Is Attenuated by Inhibition of ADAM8 or TNF

**Figure 3:**
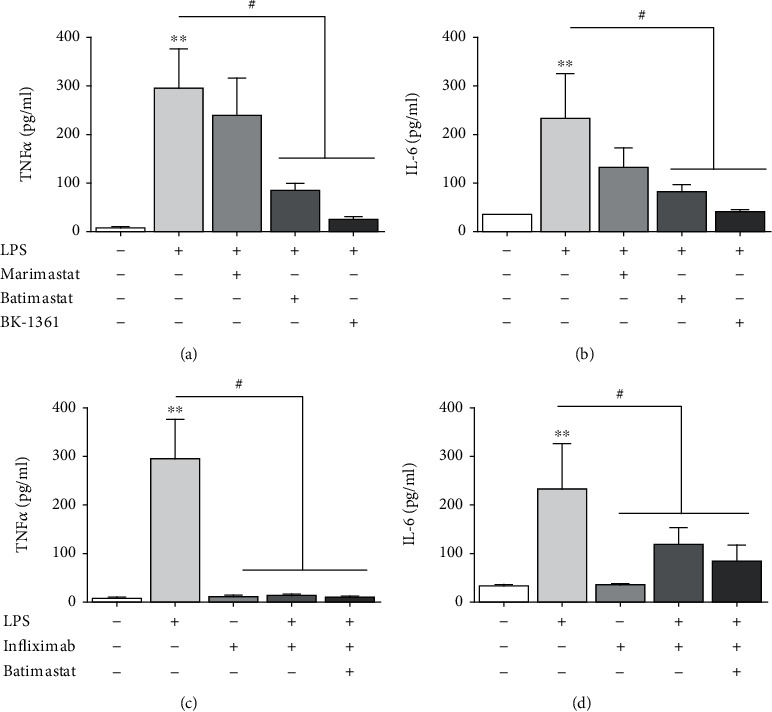
Reduction of LPS-induced cytokine release by inhibition of ADAM8 or TNF. (a, b) Murine Hepa1-6 hepatoma cells were left untreated or treated as indicated with LPS (1 *μ*g/ml) and metalloproteinase inhibitors marimastat (500 nM), batimastat (500 nM), and BK-1361 (10 *μ*g/ml) or DMSO as control. After 24 h, supernatants were collected and analysed for the release of TNF-*α* and IL-6. (c, d) Hepa1-6 cells were treated as indicated with LPS, infliximab, batimastat, or respective controls. After 24 h, supernatant was collected and analysed for the release of TNF-*α* and IL-6. Quantitative data are shown as the mean + SD of 3-4 independent experiments. Significant differences to the unstimulated controls are indicated by asterisks (^∗^*p* < 0.05, ^∗∗^*p* < 0.01, and ^∗∗∗^*p* < 0.001). Significant differences caused by the inhibitors are indicated by hashtags (^#^*p* < 0.05).

The above observations prompted us to study whether ADAM8 could play a role in LPS-induced inflammatory cytokine production. Hepatoma cells (HepG2 and Hepa1-6) were stimulated with LPS in the presence of metalloproteinase inhibitors with different selectivity and potency for ADAM8 (Figures 3(a) and 3(b) and Figures S2A and S2B). The hydroxamate-based small molecule inhibitor marimastat which is only a weak inhibitor of ADAM8 showed a minor effect, while batimastat which is a more potent hydroxamate inhibitor of ADAM8 [30] yielded a stronger inhibition. Finally, the cyclic peptide BK-1361, which is the most selective ADAM8 inhibitor and prevents the essential ADAM8 multimerisation [31], suppressed the induction and release of TNF-*α* and IL-6 most effectively. The suppression of TNF-*α* release may be a direct result of ADAM8 inhibition since membrane-bound TNF-*α* has been described to undergo ADAM8-mediated cleavage [32]. However, this would not explain the inhibition of IL-6 release. To test whether the latter effect is due to suppression of TNF-*α* release, we applied the TNF-*α* inhibitory antibody infliximab. As expected, infliximab prevented the detection of released TNF-*α*. In addition, infliximab could partially suppress IL-6 release from LPS-treated Hepa1-6 cell and this was not further reduced by combination of infliximab with batimastat (Figures 3(c) and 3(d)). This observation is consistent with previous reports showing that induction of TNF-*α* can boost LPS responses via an autocrine feedback loop dependent on TNF-*α* cleavage [33]. For HepG2 cells, only a minor reduction of IL-6 release by infliximab was observed (Figures S2C and S2D) indicating that the contribution of TNF-*α* to the LPS response may vary among cell lines.

### 3.4. LPS-Induced Cytokine Release Is Attenuated by ADAM8 Knockout in Primary Hepatocytes

**Figure 4:**
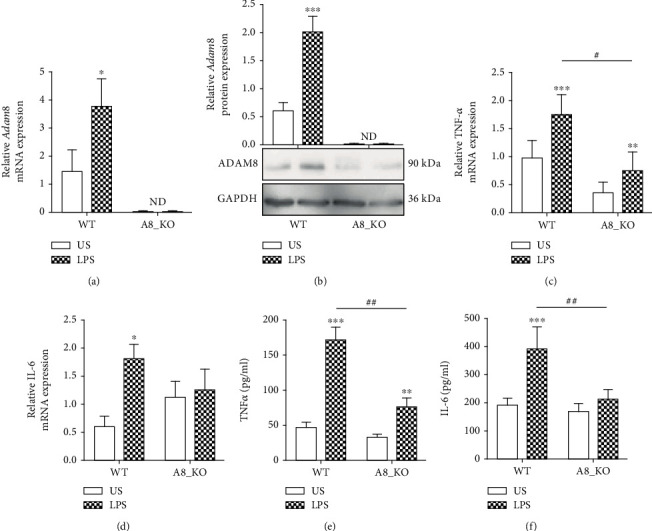
Reduction of LPS-induced cytokine expression and release in primary hepatocytes with ADAM8 knockout. (a–d) Primary murine hepatocytes were generated from wild-type (WT) mice or ADAM8 knockout (A8_KO) mice. Subsequently, cells were stimulated with or without LPS (1 *μ*g/ml) for 24 h and analysed for mRNA expression (a) and protein expression (b) of ADAM8. The data are shown as representative blot. The band intensities were quantified by densitometry and normalised to GAPDH protein expression. The mRNA expression of TNF*-α* (c) and *IL-6* (d) was also analysed by qPCR, and all qPCR data was normalised to the mRNA expression of *Rps29*. (e, f) Release of TNF-*α* and IL-6 in primary hepatocytes was analysed by ELISA. Quantitative data are shown as the mean + SD of 3-4 independent experiments. Significant differences to the unstimulated controls are indicated by asterisks (^∗^*p* < 0.05, ^∗∗^*p* < 0.01, and ^∗∗∗^*p* < 0.001). Differences caused by knockout of ADAM8 compared to control are indicated by hashtags (^#^*p* < 0.05, ^##^*p* < 0.01, and ^###^*p* < 0.001).

To demonstrate the relevance of ADAM8 for the cytokine response under more physiologic conditions, primary hepatocytes were isolated from wild-type and ADAM8 knockout mice. As shown by qPCR and Western blotting, ADAM8 was induced in wild-type primary hepatocytes by LPS treatment for 24 h on mRNA and protein level (Figures 4(a) and 4(b)). By contrast, the cells from the knockout mice completely lacked ADAM8 expression. Moreover, the LPS-induced mRNA expression of TNF-*α* and IL-6 was considerably reduced in ADAM8 knockout hepatocytes compared to the wild-type controls (Figures 4(c) and 4(d)). In line with this, the released amounts of TNF-*α* and IL-6 from LPS-stimulated cells were decreased by knockout of ADAM8 (Figures 4(e) and 4(f)). These findings indicate that ADAM8 expression is required for an efficient cytokine response of LPS-stimulated hepatocytes.

### 3.5. ADAM8 Is Overexpressed in a Murine Model of High-Fat Diet-Induced Steatohepatitis

**Figure 5:**
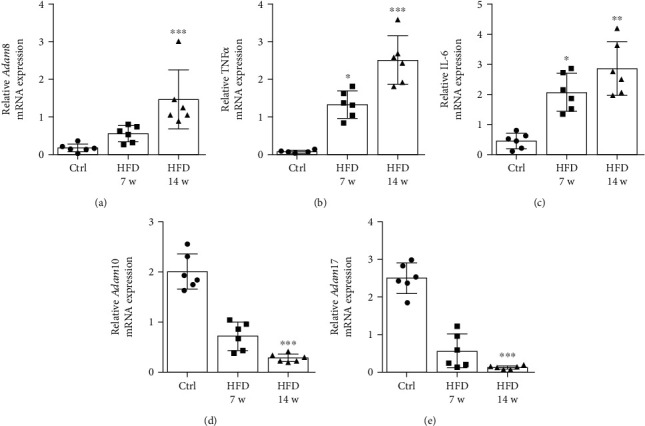
Upregulation of ADAM8 expression in the livers of mice fed on a high-fat diet. (a–e) Relative mRNA expression levels of *Adam8* (a), TNF-*α* (b), *IL-6* (c), *Adam10* (d), and *Adam17* (e) were analysed in the livers of mice that were either fed on a normal diet (Ctrl) or a high-fat diet for 7 weeks (HFD-7 w) or 14 weeks (HFD-14 w). The mRNA expression of the genes of interest was normalised to the average of three reference genes: *18S*, *β-actin*, and *Rps29*. Data are shown as the mean ± SD (*n* = 6/group). Differences to the control group are indicated by asterisks (^∗^*p* < 0.05, ^∗∗^*p* < 0.01, and ^∗∗∗^*p* < 0.001).

We next thought to transfer our insights to a model of chronic liver injury. We studied liver tissue from mice undergoing nonalcoholic fatty liver disease (NAFLD) since ADAM8 has not yet been investigated in this model. Liver tissues were obtained from mice that had been fed on a high-fat diet (HFD) for 7 and 14 weeks and had been shown to develop the phenotype similar to NAFLD especially after 14 weeks of HFD [18]. These samples were analysed for the mRNA expression of ADAM8, 10, and 17 along with the expression of the key proinflammatory cytokines TNF-*α* and IL-6 (Figures 5(a)–5(e)). The mRNA expression of ADAM8 was clearly increased in the livers at 14 weeks of HFD compared to the livers of control mice (Figure 5(a)). As expected, the mRNA expression of the key proinflammatory cytokines TNF-*α* and IL-6 was profoundly upregulated which confirms an inflammatory reaction in the livers of mice fed on HFD even after 7 weeks (Figures 4(b) and 4(c)). Noteworthy, the expression of ADAM10 and 17 was found to be significantly decreased at 14 weeks of HFD (Figures 5(d) and 5(e)).

### 3.6. Mediators of Steatohepatitis Promote ADAM8 Expression in Hepatoma, Endothelial, and Stellate Cells

**Figure 6:**
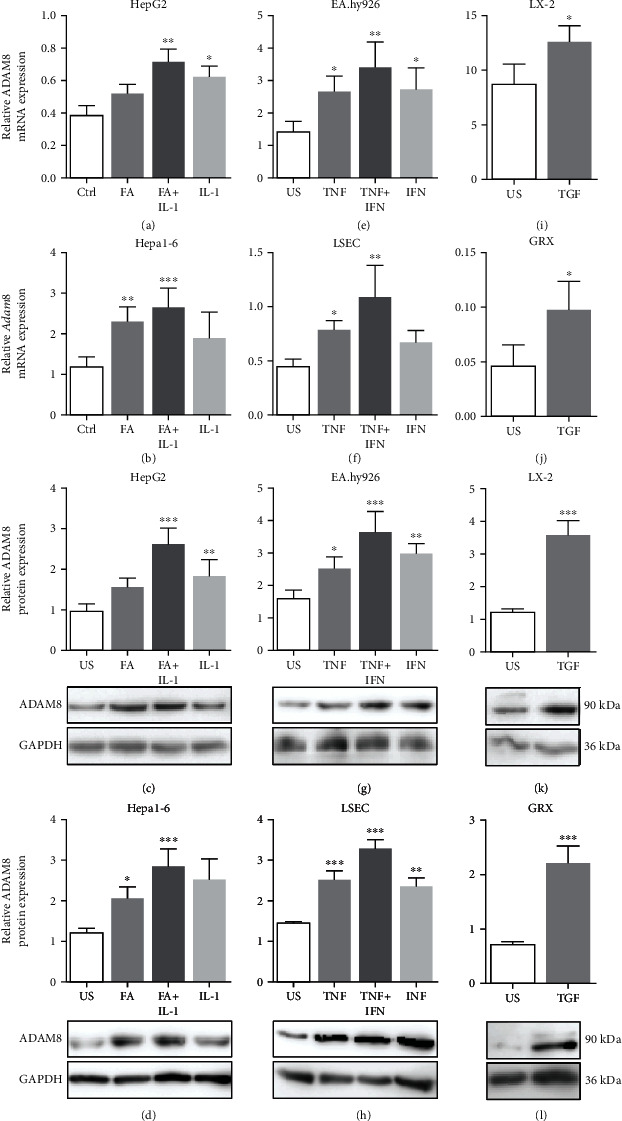
ADAM8 upregulation in cultured liver cells by mediators of steatohepatitis. (a–d) Murine Hepa1-6 and human HepG2 hepatoma cells were either left unstimulated (US) or stimulated with fatty acid (FA), IL-1*β*, or a combination of both. After 24 h, the cells were analysed for ADAM8 mRNA expression(a, b) and protein expression (c, d). (e–h) Murine LSEC and human EA.hy926 endothelial cells were left unstimulated (US) or stimulated with TNF-*α*, IFN-*γ*, or a combination of both (TI). After 24 h, the cells were analysed for ADAM8 mRNA (e, f) and protein expression (g, h). (i–l) Murine GRX and human LX-2 stellate cells were left unstimulated (US) or stimulated with TGF-*β*. After 24 h, the cells were analysed for ADAM8 mRNA (i, j) and protein expression (k, l). The mRNA expression was analysed by qPCR and normalised to *Rps29* for murine cells and to GAPDH for human cell lines. The protein expression was analysed by Western blot, and representative blots are shown for each cell line. The protein expression was quantified by densitometry and normalised to GAPDH. Quantitative data are shown as the mean + SD of 3-4 independent experiments. Significant differences to the unstimulated controls are indicated by asterisks (^∗^*p* < 0.05, ^∗∗^*p* < 0.01, and ^∗∗∗^*p* < 0.001).

To further study whether the expression levels of ADAM8 can be regulated under conditions of NAFLD *in vitro*, HepG2 and Hepa1-6 cells were treated with fatty acids (a mixture of oleic acid and linoleic acid) and interleukin-1*β* (IL-1*β*) alone or in combination to mimic the fatty liver condition [18]. After 24 h of stimulation, the cells were analysed for the expression of ADAM8. Fatty acids alone induced the expression of ADAM8 at mRNA and protein level in hepatoma cells. Moreover, the induction became more pronounced when fatty acids were used in combination with IL-1*β* (Figures 6(a)–6(d)).

Next, human EA.hy926 and murine LSEC endothelial cell lines were analysed for the regulation of ADAM8 expression under inflammatory conditions. These cells were stimulated with either TNF-*α* or interferon-gamma (IFN-*γ*) or with both [27]. After 24 h, the cell lysates were investigated for ADAM8 mRNA and protein expression. ADAM8 mRNA expression was significantly induced by either cytokine, but the most prominent effect was seen when the cells were treated with both cytokines in combination. The protein expression of ADAM8 was also increased in the same way in both cell lines (Figures 6(e)–6(h)).

Hepatic stellate cells are usually present in an inactive state and become activated when they come into contact with transforming growth factor-beta (TGF-*β*) in the course of fibrosis [34]. In the present study, human and murine hepatic stellate cell lines (LX-2 and GRX) were incubated with TGF-*β* for 24 h and examined for the induction of ADAM8 expression. The results indicated that ADAM8 is significantly upregulated in both stellate cell lines on mRNA and protein level (Figures 6(i)–6(l)).

### 3.7. Cytokine Release by Hepatoma, Endothelial, and Stellate Cells Is Attenuated by ADAM8 Knockdown

**Figure 7:**
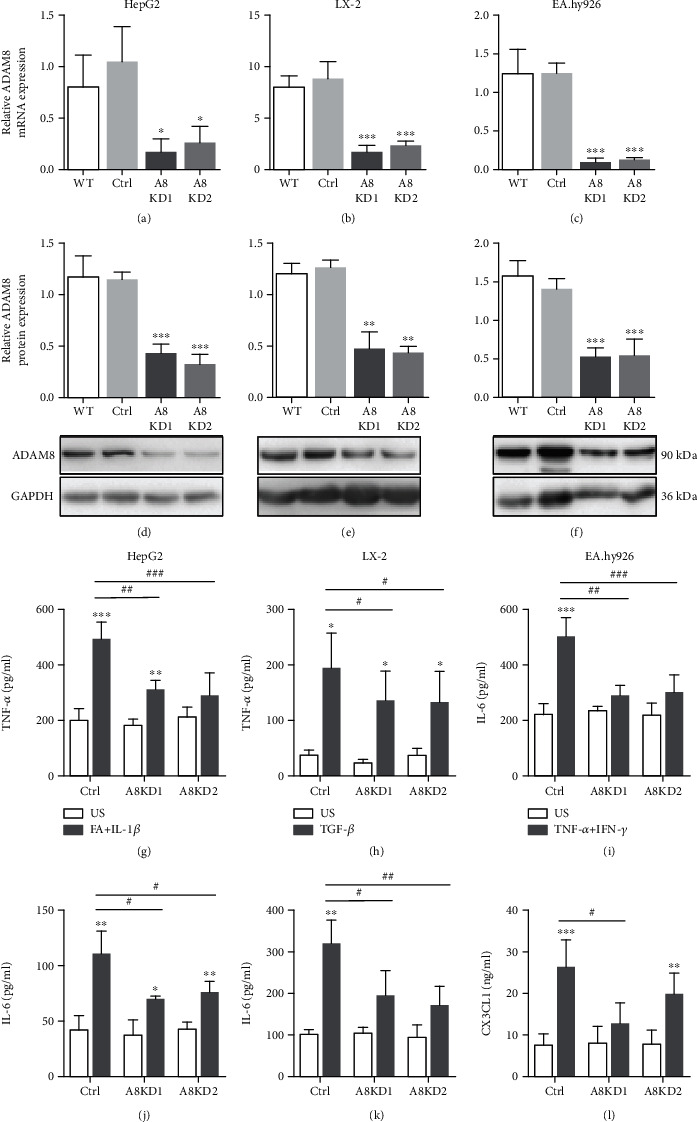
Downregulation of cytokine and chemokine release by ADAM8 knockdown. (a–f) The indicated types of human liver cells were left untransduced or transduced with lentiviruses coding for two different sequences of shRNA against ADAM8 expression (A8KD1 and A8KD2) or with control shRNA (Ctrl) to knockdown the ADAM8 expression. After 72 h, knockdown of ADAM8 was controlled on mRNA (a–c) and protein level (d–f). ADAM8 mRNA expression was quantified by qPCR and normalised to the expression of GAPDH for human cells. ADAM8 protein was detected by Western blot analysis and shown as representative blot for each cell line (d–f). The band intensities were quantified by densitometry and normalised to that of GAPDH. (g–l) The indicated types of cells with or without ADAM8 knockdown were left unstimulated (US) or stimulated with the indicated mediators. After 24 h, concentrations of released cytokines (TNF-*α* and IL-6) and chemokines (CX3CL1) in the supernatants were determined by ELISA. Quantitative data are shown as the mean + SD of 3-4 independent experiments, and representative Western blots are shown. Significant differences caused by cell stimulation are indicated by asterisks (^∗^*p* < 0.05, ^∗∗^*p* < 0.01, and ^∗∗∗^*p* < 0.001), and differences caused by knockdown of ADAM8 compared to control are indicated by hashtags (^#^*p* < 0.05, ^##^*p* < 0.01, and ^###^*p* < 0.001).

The upregulation of ADAM8 in *in vivo* and *in vitro* models raised the question of whether ADAM8 modulates the inflammatory response under conditions of NAFLD. For this purpose, the expression of ADAM8 was silenced in human cells by transduction with lentivirus coding for shRNA against ADAM8 and in murine cells by transfection of siRNA against ADAM8. The silencing of ADAM8 was confirmed at gene and protein levels *via* qPCR and Western blot analysis, respectively (Figures 7(a)–7(f) and Figures S3A–S3F). Furthermore, we tested the effect of ADAM8 knockdown on the cytokine release by stimulated hepatoma, endothelial, and stellate cell lines. For stimulation of hepatoma cells, a combination of fatty acid and IL-1*β* was used. The endothelial cells were stimulated with a combination of TNF-*α* and IFN-*γ*, while stellate cells received TGF-*β* to induce cytokine and chemokine release. As determined by ELISA, knockdown of ADAM8 expression attenuated the release of TNF-*α* and IL-6 by hepatoma cells and stellate cells as well as the release of IL-6 and CX3CL1 by endothelial cells (Figures 7(g)–7(l) and Figures S4G–S4L). Notably, this effect was not seen for all tested inflammatory mediators. For example, release of the chemokine CXCL16 by stimulated murine (Figures S4A–S4C) and human (Figures S4G–S4I) hepatoma, endothelial, or stellate cells remained unaffected. The release of murine CXCL1 (KC, keratinocyte-derived chemokine) was found attenuated by ADAM8 knockdown in murine hepatoma and endothelial cells but not in stellate cells (Figures S5D–S5F). Moreover, the release of human CXCL8 (IL-8) was only attenuated in human hepatoma cells but not in endothelial or stellate cells by ADAM8 knockdown (Figures S4J–S4L). Thus, the requirement of ADAM8 for the inflammatory response seems to depend on the type of mediator released from a given cell type.

## 4. Discussion

Acute and chronic liver inflammation is driven by cytokine and chemokine release from various cell types in the liver [5, 6]. Here, we demonstrate that the induction of inflammatory mediators is correlated with an increased expression of ADAM8 in hepatic cells, endothelial cells, and stellate cells. By knockdown and knockout of ADAM8 expression, we provide double evidence that ADAM8 expression is critical for an effective induction of TNF-*α* and IL-6 mRNA and protein expression. To our knowledge, this novel function of ADAM8 has not been reported before. Inhibition studies suggest that the proteolytic activity of ADAM8 and signalling by TNF-*α* can contribute to these ADAM8-dependent effects.

While the related proteases ADAM10 and ADAM17 are constitutively expressed and critical in many cells and tissues during development and also in healthy adult organisms, the expression of ADAM8 is only sparse. During later stages of the development, ADAM8 expression becomes upregulated in distinct cell types of the organism but, due to compensatory mechanisms, knockout of ADAM8 did not lead to developmental defects [35]. In accordance, the livers of ADAM8-deficient mice appeared normal. In these mice, we found a very low basal expression of ADAM8 in the liver of wild-type mice which corresponded to low ADAM8 expression in different human or murine liver cell types, including hepatocytes, sinusoidal endothelial cells, and stellate cells. The ADAM8 knockout mice (8 weeks old) used for histology and for generation of primary hepatocytes in this study were healthy and did not show obvious alterations in the liver, and liver enzyme levels were normal. In agreement, viability and morphology of primary hepatocytes were also not affected by ADAM8 deficiency. We therefore conclude that under basal conditions, ADAM8 is expressed at low levels and does not seem to have critical functions in liver homeostasis.

In a model of acute liver inflammation, we observed that ADAM8 is markedly upregulated by LPS on the mRNA and protein level. Also *in vitro*, ADAM8 expression is upregulated by LPS in hepatocyte, endothelial, and stellate cells. In fact, induction of ADAM8 by proinflammatory stimuli including LPS and TNF-*α* has been previously reported for monocytes, neuronal cells, astrocytes, and microglia [36]. In a previous study, we observed ADAM8 upregulation in LPS-induced lung inflammation and found ADAM8 induction by LPS in cultured lung endothelial cells and leukocytes [13]. We therefore propose that ADAM8 is part of the acute inflammatory response machinery. It has been demonstrated that ADAM8 on leukocytes promotes cell migration and this activity can be essential for the inflammatory response in the lung. This has been demonstrated with ADAM8 knockout mice by showing reduced leukocyte recruitment and cytokine expression when challenged intranasally with LPS [13]. In the present study, we show that ADAM8 expression is also required for the mRNA induction and protein expression of inflammatory cytokines in response to LPS. This additional activity may be even more relevant for the inflammatory response especially in the liver. Further *in vivo* studies are required to find out whether ADAM8 deficiency can protect mice from inflammatory cytokine induction upon LPS challenge.

Also in chronic models of liver inflammation including nonalcoholic steatohepatitis, we observed increased ADAM8 expression. Thus, ADAM8 upregulation is not only part of the acute response but is additionally involved in chronic liver inflammation. A previous study already showed increased ADAM8 expression in biopsies of patients with chronic liver inflammation [37]. In the present study, we describe mediators involved in steatohepatitis that induce ADAM8 expression. ADAM8 is induced by a combination of IL-1*β* and fatty acid in hepatocytes, a combination of TNF-*α* and IFN-*γ* in endothelial cells and by the profibrotic cytokine TGF-*β* in stellate cells. In fact, in human biopsies, increased expression of ADAM8 was localised to hepatic stellate cells [37]. Apart from the liver, ADAM8 upregulation has been described for other chronic inflammatory diseases such as asthma [11, 12], COPD [14], and atherosclerosis [38, 39]. Of note, for these diseases, proinflammatory as well as protective functions of ADAM8 have been reported. Different studies found that ADAM8 knockout or inhibition can attenuate asthma [10, 12]. Along with this, a reduction of inflammatory chemokines has been observed. By contrast, others have reported that ADAM8 knockout exacerbates asthma or COPD by enhancing apoptosis [14]. Yet, with regard to steatohepatitis or other chronic inflammatory liver diseases, the role of ADAM8 is unclear and needs to be investigated. In this situation, the contribution of immune cells can be detrimental or protective and the impact of immune cells in a certain pathological situation might determine the role of ADAM8.

ADAM8 can act as a proteolytic enzyme via its metalloproteinase domain. Using two hydroxamate-based inhibitors with different selectivity for the protease activity of ADAM8 [30], we obtained initial evidence that ADAM8 activity can contribute to the induced cytokine production in hepatoma cells. Several substrates have been described for ADAM8 including TNF-*α*, EGFR ligands, and CX3CL1 [32]. Cleavage of these substrates may lead to the generation of soluble ligands that promote autocrine stimulation. In fact, TNF-*α* is a well-known inducer of acute liver inflammation. In our experiments, we observed clear reduction of LPS-induced TNF-*α* release upon ADAM8 inhibition. Moreover, we found that TNF-*α* inhibition similar to ADAM8 inhibition suppressed IL-6 release. These findings support the model that LPS-induced and ADAM8-dependent release of TNF-*α* could lead to autocrine stimulation and in turn boosts the induction of IL-6 and further upregulates TNF-*α* expression. A similar amplification loop via TNF cleavage has been recently reported for LPS-stimulated macrophages [33]. In these cells, most of the TNF-*α* cleavage is attributed to ADAM17. In addition to ADAM17, ADAM8 has been reported to cleave TNFR-1 and generates a soluble antagonist. This may indicate a negative feedback loop in which ADAM17- and ADAM8-dependent induction and release of TNF-*α* is again counterbalanced by TNFR shedding [36]. Whether cleavage of other possible ADAM8 substrates such as the EGFR ligand amphiregulin or the chemokine CX3CL1 which are critically implicated in chronic liver inflammation can also be relevant in the setup of our study remains to be determined [37].

Besides its proteolytic function, ADAM8 can interact via its disintegrin domain with integrins such as *β*1- and *β*3-integrin, which are coexpressed on the cell membrane [31]. Of note, the peptide inhibitor BK-1361 can block this interaction [31]. Thus, the inhibition data do not rule out that part of the ADAM8-dependent effect in our setup is mediated via the integrin pathway. ADAM8 is known to promote *β*3-integrin-dependent activation of focal adhesion kinase, Scr kinase, mitogen-activated protein kinase, and Rho A GTPase [16]. This interaction seems to be crucial for the promigratory function of ADAM8 that has been observed in leukocytes and in various cancer cells including hepatoma cells [13, 16]. Interestingly, *β*3-integrin signalling via FAK and Src can promote activation of the transcription factor NF-*κ*B [40]. It is therefore still possible that activation of these kinases via the protease-independent activity of ADAM8 synergistically enhances the LPS or cytokine-induced activation of the NF-*κ*B pathway leading to expression of further proinflammatory mediators and effector molecules.

Our finding that ADAM8 expression is upregulated under inflammatory conditions *in vivo* and *in vitro* and required for effective inflammatory cytokine production of liver cells raises important further questions about the use of ADAM8 as an inflammatory marker in liver biopsies of patients, the contribution to inflammatory liver diseases, and finally the suitability of ADAM8 as a therapeutic target. When developing and testing inhibition approaches against ADAM8, the ability of the protease to promote an inflammatory cytokine response should be considered.

## 5. Conclusion

Here, we demonstrate that the induction of inflammatory mediators during acute and chronic liver inflammation is correlated with an increased expression of ADAM8. Increased ADAM8 expression is critical for effective induction of TNF-*α* and IL-6 expression in hepatocyte, endothelial, and stellate cells. Inhibition studies suggest that the proteolytic activity of ADAM8 is involved in boosting the cytokine response and that TNF-*α* is induced and released in an ADAM8-dependent manner causing autocrine stimulation of further cytokine production. This novel function of ADAM8 may place ADAM8 as a critical promoter of the inflammatory response in acute and chronic liver diseases. The pathogenic role of ADAM8 may depend on the contribution of the type of inflammatory response to the disease and this needs to be further investigated in animal models.

## Data Availability

The original datasets generated or analysed during the present study are available from the corresponding author on reasonable request.

## Supplementary Materials

Supplementary Materials Table S1: qPCR for targeted genes was run for 40 cycles of 10 s denaturation at 95°C, followed by 30 s annealing at indicated temperatures and 15 s amplification at 72°C. Primer sequences used in this study are listed below. Figure S1: mRNA expression of ADAM8 in liver tissues of LPS-treated mice. A: wild-type (WT) mice were treated with 0.9% NaCl or with LPS (750 *μ*g/kg) for 6 h. Subsequently, mice were sacrificed and liver tissues were collected from each group. These tissues were analysed for mRNA expression of *Adam8* by qPCR. The mRNA expression of *Adam8* was normalised to the mRNA expression of reference gene *Gapdh*. Significant differences are indicated by asterisks (^∗^*p* < 0.05, ^∗∗^*p* < 0.01, and ^∗∗∗^*p* < 0.001). Figure S2: reduction of LPS-induced cytokine release by inhibition of ADAM8 or TNF. A, B: human HepG2 hepatoma cells were treated with DMSO as a control, LPS (1 *μ*g/ml), and the metalloproteinase inhibitors marimastat (BB-2516) (500 nM), batimastat (BB-94) (500 nM), and BK-1361 (10 *μ*g/ml). After 24 h, supernatants were collected and analysed for the release of TNF-*α* and IL-6. C, D: HepG2 cells were treated as indicated with LPS, infliximab, batimastat, or respective controls. After 24 h, supernatant was collected and analysed for the release of TNF-*α* and IL-6. Quantitative data are shown as the mean + SD of 3-4 independent experiments. Significant differences compared to the control are indicated by asterisks (^∗^*p* < 0.05, ^∗∗^*p* < 0.01, and ^∗∗∗^*p* < 0.001). Figure S3: downregulation of cytokine and chemokine release by ADAM8 knockdown in murine liver cells. A–F: the indicated types of murine liver cells were left untransfected or transfected with two different sequences of siRNA against ADAM8 expression (A8KD1 and A8KD2) or control siRNA (Ctrl). After 72 h, knockdown of ADAM8 was controlled on mRNA and protein level. *Adam8* mRNA expression was quantified by qPCR and normalised to the expression of *Rps29* for murine cells. ADAM8 protein expression was detected by Western blot analysis and shown as representative blot for each cell line. Protein bands were separated by a line when they were run on the same blot but not side by side. The band intensities were quantified by densitometry and normalised to that of GAPDH. G–L: the indicated types of cells with or without ADAM8 knockdown were left unstimulated (US) or stimulated with the indicated mediators. After 24 h, concentrations of released cytokines (TNF-*α* and IL-6) and chemokines (CX3CL1) in the supernatants were determined by ELISA. Data are shown as the mean + SD and representative of 3-4 independent experiments. Significant differences caused by cell stimulation are indicated by asterisks (^∗^*p* < 0.05, ^∗∗^*p* < 0.01, and ^∗∗∗^*p* < 0.001), and differences caused by knockdown of ADAM8 compared to control are indicated by hashtags (^#^*p* < 0.05, ^##^*p* < 0.01, and ^###^*p* < 0.001). Figure S4: effect of ADAM8 knockdown on the LPS-induced release of other inflammatory mediators. A–F: the indicated types of murine liver cells were left untransfected or transfected with two different sequences of siRNA against ADAM8 expression (A8KD1 and A8KD2) or control siRNA (Ctrl). Cells were then left unstimulated (US) or stimulated with the indicated mediators. After 24 h, concentrations of released CXCL16 and KC in the supernatants were determined by ELISA. G–L: the indicated types of human liver cells were left untransduced or transduced with lentiviruses coding for two different sequences of shRNA against ADAM8 expression (A8KD1 and A8KD2) or with control shRNA (Ctrl). Cells were then left unstimulated (US) or stimulated with the indicated mediators. After 24 h, concentrations of released CXCL16 and IL-8 in the supernatants were determined by ELISA. Data are shown as the mean + SD and representative of 3-4 independent experiments. Significant differences caused by cell stimulation are indicated by asterisks (^∗^*p* < 0.05, ^∗∗^*p* < 0.01, and ^∗∗∗^*p* < 0.001), and differences caused by knockdown of ADAM8 compared to control are indicated by hashtags (^#^*p* < 0.05, ^##^*p* < 0.01, and ^###^*p* < 0.001).
