# Supplementary material for: Expression of the Metalloproteinase ADAM8 Is Upregulated in Liver Inflammation Models and Enhances Cytokine Release In Vitro
Source: Mediators Inflamm. 2021 Mar 11;2021:6665028. doi: 10.1155/2021/6665028 (PMC7987468; doi:10.1155/2021/6665028)
Supplement: Supplementary Materials — Table S1: qPCR for targeted genes was run for 40 cycles of 10 s denaturation at 95°C, followed by 30 s annealing at indicated temperatures and 15 s amplification at 72°C. Primer sequences used in this study are listed below. Figure S1: mRNA expression of ADAM8 in liver tissues of LPS-treated mice. A: wild-type (WT) mice were treated with 0.9% NaCl or with LPS (750 μg/kg) for 6 h. Subsequently, mice were sacrificed and liver tissues were collected from each group. These tissues were analysed for mRNA expression of Adam8 by qPCR. The mRNA expression of Adam8 was normalised to the mRNA expression of reference gene Gapdh. Significant differences are indicated by asterisks (∗p < 0.05, ∗∗p < 0.01, and ∗∗∗p < 0.001). Figure S2: reduction of LPS-induced cytokine release by inhibition of ADAM8 or TNF. A, B: human HepG2 hepatoma cells were treated with DMSO as a control, LPS (1 μg/ml), and the metalloproteinase inhibitors marimastat (BB-2516) (500 nM), batimastat (BB-94) (500 nM), and BK-1361 (10 μg/ml). After 24 h, supernatants were collected and analysed for the release of TNF-α and IL-6. C, D: HepG2 cells were treated as indicated with LPS, infliximab, batimastat, or respective controls. After 24 h, supernatant was collected and analysed for the release of TNF-α and IL-6. Quantitative data are shown as the mean + SD of 3-4 independent experiments. Significant differences compared to the control are indicated by asterisks (∗p < 0.05, ∗∗p < 0.01, and ∗∗∗p < 0.001). Figure S3: downregulation of cytokine and chemokine release by ADAM8 knockdown in murine liver cells. A–F: the indicated types of murine liver cells were left untransfected or transfected with two different sequences of siRNA against ADAM8 expression (A8KD1 and A8KD2) or control siRNA (Ctrl). After 72 h, knockdown of ADAM8 was controlled on mRNA and protein level. Adam8 mRNA expression was quantified by qPCR and normalised to the expression of Rps29 for murine cells. ADAM8 protein expression was detected by Wester [file 6665028.f1.docx]

**Expression of the metalloproteinase ADAM8 is up-regulated in liver inflammation models and enhances cytokine release *in vitro***

Tanzeela Awan^1^, Aaron Babendreyer^1^, Justyna Wozniak^1^, Abid Mahmood Alvi^1^, Viktor Sterzer^2^, Lena Cook^3^, Jörg W. Bartsch^3^, Christian Liedtke^4^, Daniela Yildiz^5^, Andreas Ludwig^1*^

^1^Institute of Molecular Pharmacology, RWTH Aachen University, Aachen, Germany

^2^Department of Medicine II, University Hospital RWTH Aachen, Aachen, Germany

^3^Department of Neurosurgery, Philipps University Marburg, University Hospital Marburg, Marburg, Germany

^4^Department of Medicine III, University Hospital RWTH Aachen, Aachen, Germany

^5^Institute of Experimental and Clinical Pharmacology and Toxicology, PZMS, ZHMB, Saarland University, Homburg, Germany

**Running title:** ADAM8 in liver inflammation

*Address correspondence to:

Andreas Ludwig,

Institute of Pharmacology and Toxicology,

RWTH Aachen University, Pauwelsstr. 30, 52074 Aachen, Germany.

Phone: +49 241 8035771, Fax: +49 241 8082433, E-Mail: aludwig@ukaachen.de

**Supplementary Figures:**

**
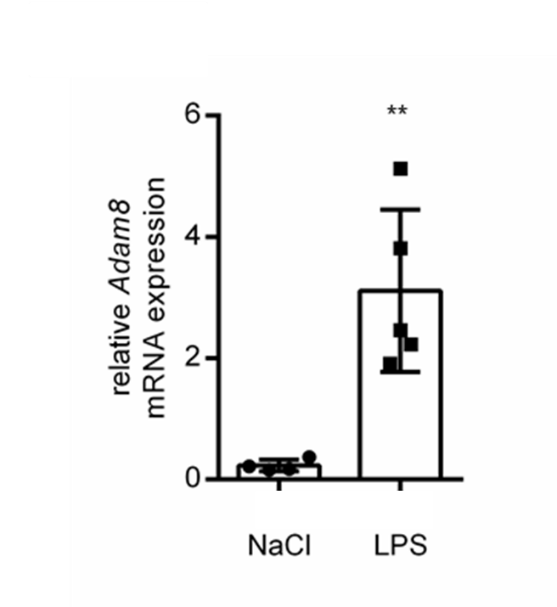
**

**Figure S1: mRNA expression of ADAM8 in liver tissues of LPS-treated mice**

**A:** Wildtype (WT) mice were treated with 0.9% NaCl or with LPS (750μg/kg) for 6 h. Subsequently, mice were sacrificed and liver tissues were collected from each group. These tissues were analysed for mRNA expression of *Adam8* by qPCR. The mRNA expression of *Adam8* was normalised to the mRNA expression of reference gene *Gapdh*. Significant differences are indicated by asterisks (* p< 0.05, ** p<0.01, *** p<0.001)

**
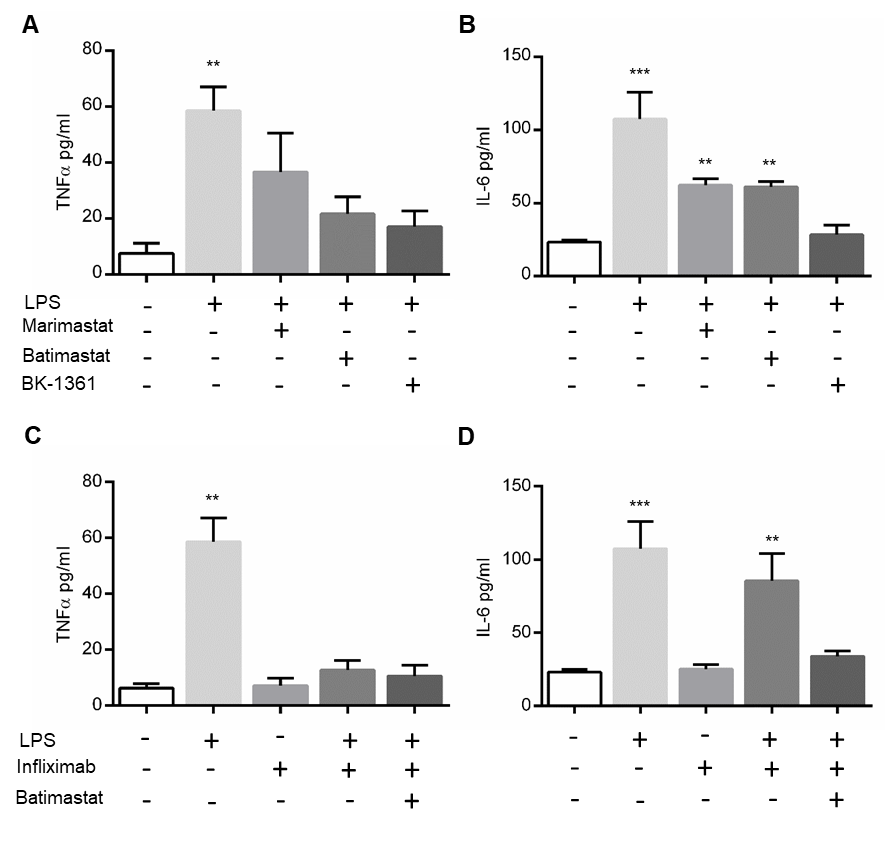
**

**Figure S2: Reduction of LPS induced cytokine release by inhibition of ADAM8 or TNF**

**A-B:** Human HepG2 hepatoma cells were treated with DMSO as a control, LPS (1µg/ml) and the metalloproteinase inhibitors marimastat (BB-2516) (500nM), batimastat (BB-94) (500nM) and BK-1361 (10µg/ml). After 24h, supernatants were collected and analysed for the release of TNFα and IL-6. **C-D:** HepG2 cells were treated as indicated with LPS, infliximab, batimastat or respective controls. After 24h, supernatant was collected and analysed for the release of TNFα and IL-6. Quantitative data are shown as mean + SD of 3-4 independent experiments. Significant differences compred to the control are indicated by asterisks* p< 0.05, ** p<0.01, *** p<0.001.


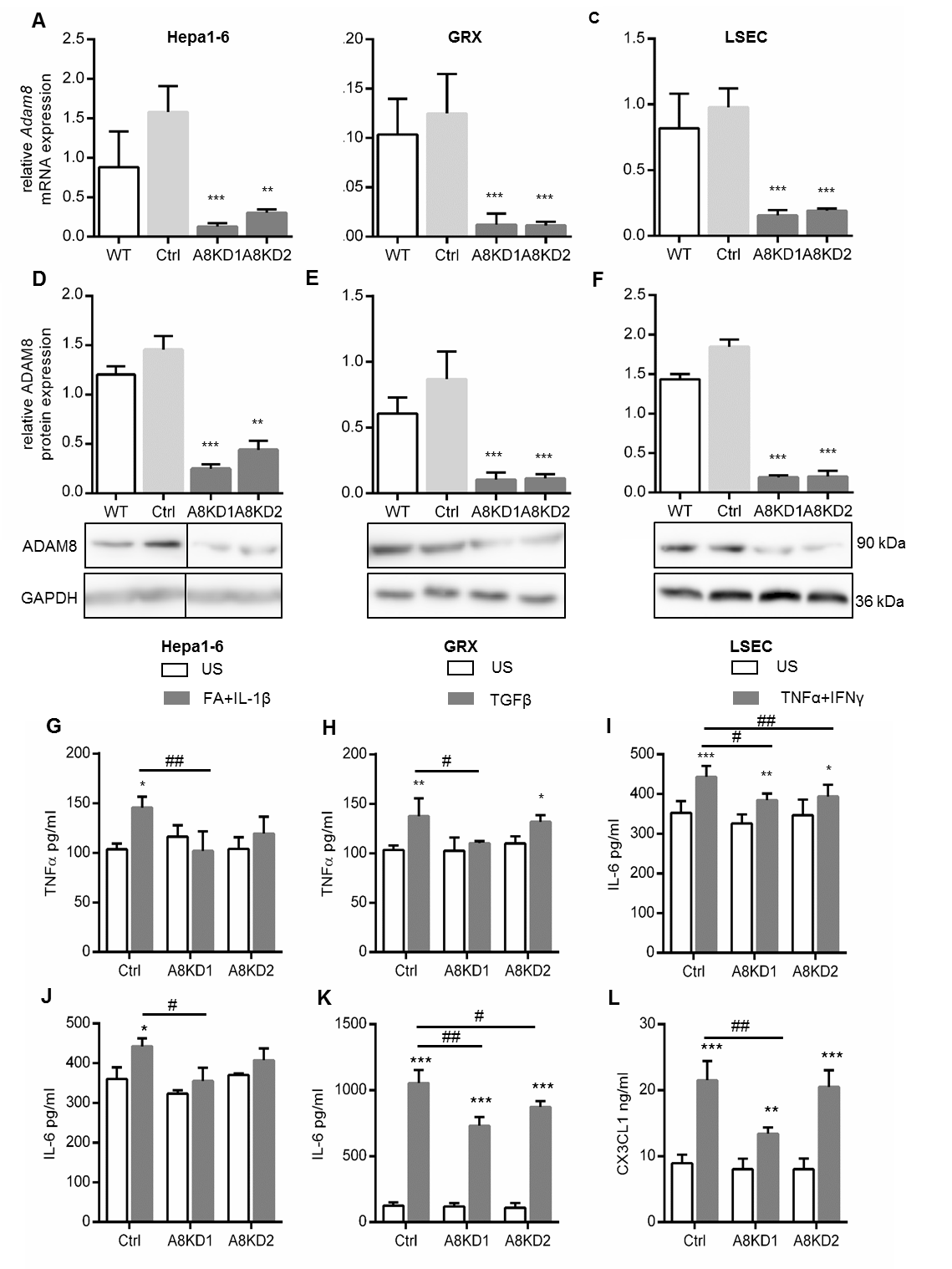


**Figure S3: Down-regulation of cytokine and chemokine release by ADAM8 knockdown in murine liver cells**

**A-F:** The indicated types of murine liver cells were left untransfected or transfected with two different sequences of siRNA against ADAM8 expression (A8KD1 & A8KD2) or control siRNA (Ctrl). After 72h, knockdown of ADAM8 was controlled on mRNA and protein level. *Adam8* mRNA expression was quantified by qPCR and normalised to the expression of *Rps29* for murine cells. ADAM8 protein expression was detected by Western blot analysis and shown as representative blot for each cell line. Protein bands were separated by a line when they were run on the same blot but not side by side. The band intensities were quantified by densitometry and normalised to that of GAPDH. **G-L:** The indicated types of cells with or without ADAM8 knockdown were left unstimulated (US) or stimulated with the indicated mediators. After 24h, concentrations of released cytokines (TNFα and IL-6) and chemokines (CX3CL1) in the supernatants were determined by ELISA. Data are shown as mean + SD and representative of 3-4 independent experiments. Significant differnces caused by cell stimulation are indicated by asterisks (* p< 0.05, ** p<0.01, *** p<0.001) and differences caused by knockdown of ADAM8 compared to control are indicated by hashes (# p< 0.05. ## p<0.01, ### p<0.001).

**
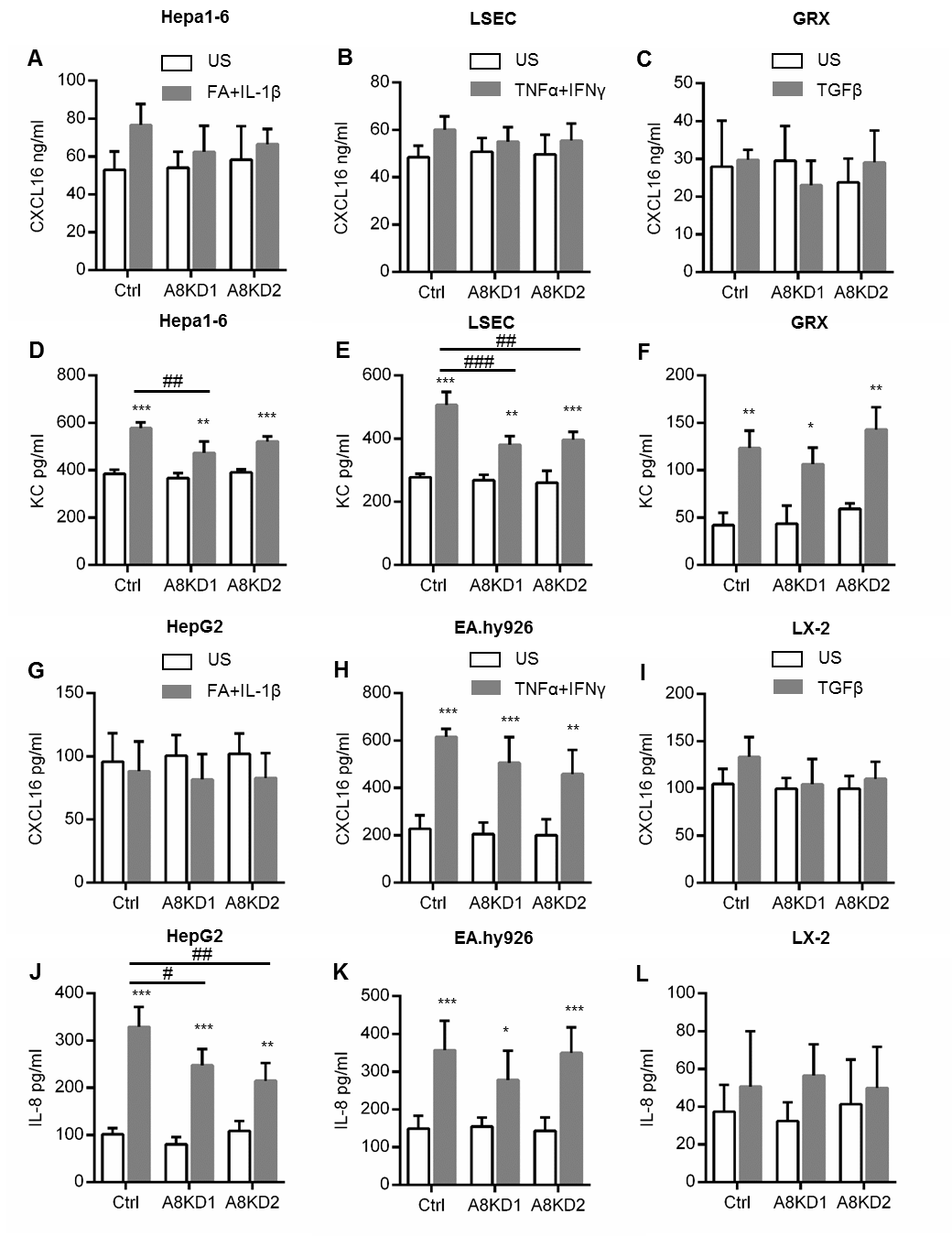
**

**Figure S4: Effect of ADAM8 knockdown on the LPS induced release of other inflammatory mediators**

**A-F:** The indicated types of murine liver cells were left un-transfected or transfected with two different sequences of siRNA against ADAM8 expression (A8KD1 & A8KD2) or control siRNA (Ctrl). Cells were then left unstimulated (US) or stimulated with the indicated mediators. After 24h, concentrations of released CXCL16 and KC in the supernatants were determined by ELISA. **G-L:** The indicated types of human liver cells were left untransduced or transduceded with lentiviruses coding for two different sequences of shRNA against ADAM8 expression (A8KD1 & A8KD2) or with control shRNA (Ctrl). Cells were then left unstimulated (US) or stimulated with the indicated mediators. After 24h, concentrations of released CXCL16 and IL-8 in the supernatants were determined by ELISA. Data are shown as mean + SD and representative of 3-4 independent experiments. Significant differnces caused by cell stimulation are indicated by asterisks (* p< 0.05, ** p<0.01, *** p<0.001) and differences caused by knockdown of ADAM8 compared to control are indicated by hashes (# p< 0.05. ## p<0.01, ### p<0.001).

**Table S1:** qPCR for targeted genes was run for 40 cycles of 10 s denaturation at 95 °C, followed by 30 s annealing at indicated temperatures and 15 s amplification at 72 °C. Primer sequences used in this study are listed below.

| **Target gene** | **Sequence 5ʹ→ 3ʹ** | **Annealing temperature ºC** |
| --- | --- | --- |
| hADAM8 | Forward: AAGCAGCCGTGCGTCATC  Reverse: AACCTGTCCTGACTATTCCAAATCTC | 62 |
| hTNFα | Forward: CCTCTCTCTAATCAGCCCTCTG  Reverse: GAGGACCTGGGAGTAAGTGAG | 59 |
| hIL-6 | Forward: ACTCACCTCTTCAGAACGAATTGC  Reverse: CCATCTTTGGAAGGTTCAGGTTG | 60 |
| hGAPDH | Forward: CCAGCCCCAGCGTCAAAGGTG  Reverse: AGGGCCGATCATGGAGTCTT | 66 |
| *mAdam10* | Forward: AGCAACATCTGGGGACAAAC  Reverse: TGGCCAGATTCAACAAAACA | 57 |
| *mAdam17* | Forward: AAACCAGAACAGACCCAACG  Reverse: AACGAATCGAACCCTGACTGGCA | 57 |
| *mAdam8* | Forward: GCGAGTGCTGGAGGTTGTAA  Reverse: ACCCCCGTGATAAGTTGCAC | 64 |
| *mRps29* | Forward: CCTTTCTCCTCGTTGGGC  Reverse: GAGCAGACGCGGCAA | 61 |
| *mTnfα* | Forward: GAA CTG GCA GAA GAG GC  Reverse: CAT AGA ACT GAT GAG AGG GAG | 61 |
| *mIl-6* | Forward: TGCAAGAGAVTTCCATCCAGTTGCC  Reverse: AAGCCTCCGACTTGTGAAGTGGT | 59 |
| *mGapdh* | Forward: GGCAATTCAACGGCACAGT  Reverse: AGATGGTGATGGGCTTCCC | 63 |
| *mβ actin* | Forward: AAGGCCAACCGTGAAAAGAT  Reverse: GTGGTACGACCAGAGGCATAC | 58 |
